# Evaluating the effectiveness of the National Health Insurance Subsidy Programme within Kenya’s universal health coverage initiative: a study protocol

**DOI:** 10.1136/bmjopen-2024-083971

**Published:** 2024-11-21

**Authors:** Beryl Maritim, Jacinta Nzinga, Benjamin Tsofa, Anita Musiega, Peter Mwangi Mugo, Ethan Wong, Caitlin Mazzilli, Wangari Ng'an'ga, Brittany Hagedorn, Gillian Turner, Anne Musuva, Felix Murira, Nirmala Ravishankar, Edwine Barasa

**Affiliations:** 1 Health Economics Research Unit, KEMRI-Wellcome Trust Research Programme Nairobi, Nairobi, Kenya; 2 KEMRI-Wellcome Trust Research Programme Nairobi, Nairobi, Kenya; 3 Health Policy and Systems Research, KEMRI-Wellcome Trust Research Programme, Kilifi, Kenya; 4 KEMRI-Wellcome Trust Research Programme, Nairobi, Kenya; 5 Bill & Melinda Gates Foundation, Seattle, Washington, USA; 6 Gates Foundation, Seattle, Washington, USA; 7 ThinkWell Global, Washington, Washington, USA; 8 ThinkWell Global, Nairobi, Kenya; 9 Health Economics Research Unit, Kenya Medical Research Institute, Nairobi, Kenya; 10 Center for Tropical Medicine and Global Health, University of Oxford Nuffield Department of Medicine, Oxford, UK

**Keywords:** HEALTH ECONOMICS, Health Equity, Health economics, Health policy

## Abstract

**Abstract:**

**Background:**

Low-income and middle-income countries, including Kenya, are pursuing universal health coverage (UHC) through the establishment of Social Health Insurance programmes. As Kenya rolls out the recently unveiled UHC strategy that includes a national indigent cover programme, the goal of this study is to evaluate the impact of health insurance subsidy on poor households’ healthcare costs and utilisation. We will also assess the effectiveness and equity in the beneficiary identification approach employed.

**Methodology and analysis:**

Using a quantitative design with quasi-experimental and cross-sectional methods, our matched cohort study will recruit 1350 households across three purposively selected counties. The ‘exposure’ arm, enrolled in the UHC indigent programme, will be compared with a control arm of eligible but unenrolled households over 12 months. Coarsened exact matching will be used to pair households based on baseline characteristics, analysing differences in expenses and catastrophic health expenditure. A cross-sectional design will be employed to evaluate the effectiveness and equity in beneficiary identification, estimating inclusion errors associated with the subsidy programme while assessing gender equity.

**Ethics and dissemination:**

Ethical approval has been obtained from the Scientific and Ethics Review Unit at Kenya Medical Research Institute, with additional permissions sought from County Health Departments. Participants will provide written informed consent. Dissemination strategies include peer-reviewed publications, conference presentations and policy-maker engagement for broad accessibility and impact.

**STRENGTHS AND LIMITATIONS OF THIS STUDY**

- The study uses a quantitative design with quasi-experimental and cross-sectional methods, which allows for a comprehensive evaluation of the equity and effectiveness of beneficiary identification and the impact of the health insurance subsidy.
- A quasi-experimental prospective matched cohort study will be conducted to evaluate the impact of the indigent programme on household expenditure and health service utilisation. The use of matching helps control for confounding variables and strengthens the internal validity of the results, making it a more rigorous alternative to standard observational designs that may suffer from selection bias.
- A cross-sectional study design will be used to evaluate the effectiveness and equity in beneficiary identification, allowing for the estimation of inclusion errors. This method enables the assessment of a large population at a single point in time, which is cost-effective and practical when assessing beneficiary identification accuracy.
- The main limitation of this study pertains to the dynamic nature of universal health coverage reforms, introducing a challenge in maintaining a static description and implementation of the intervention, and consequently the evaluation methodology.

## Background

 Low-income and middle-income countries (LMICs) have prioritised universal health coverage (UHC) and are undertaking health financing reforms to provide financial risk protection as a part of national UHC reforms.^1 2^ The establishment of social health insurance (SHI) programmes to expand the prepayment of h.ealth services is an increasingly common mechanism in African countries. These initiatives mobilise funds primarily through member contributions in the form of health insurance premiums.^3^ Countries such as Ghana, Kenya and Rwanda have implemented National Health Insurance programmes while others like South Africa and Uganda are in the process of designing and implementing SHI systems.^4^,^7^

As countries seek to scale up prepayment mechanisms, there is ongoing debate about whether countries should adopt a universal approach using tax-funded systems or a targeted approach with household contributions and subsidies for the poor.^8 9^ A universal approach aims to provide health coverage to the entire population ensuring that everyone, regardless of socioeconomic status (SES), has access to essential health services with a focus on primary healthcare.^10 11^ This approach is founded on egalitarianism that espouses equality for all people.^12^ In this approach, governments play a central role, taking responsibility for the health of the entire population. In addition, policies are designed to cover everyone using funding mechanisms such as taxation that contribute to broad financial risk pooling. Countries, such as Thailand, have effectively established a UHC programme, ensuring the provision of essential healthcare services to all citizens regardless of their SES.^13^,^15^

The targeted approach focuses on specific vulnerable or disadvantaged groups within the population, such as the poor or those with specific health needs.^11 16^ This approach is founded on libertarian views that often argue that individuals should take responsibility for their health and well-being and contribute to their healthcare expenses.^12^ Support for the universal approach emphasises its aims for equity by providing coverage for all, as opposed to a targeted approach that raises concerns about leaving some groups underserved.^17 18^ In addition, targeted approaches often involve more complex administration and eligibility criteria, whereas universal systems aim for simplicity and broad coverage. Proponents of a targeted approach, however, argue that it gives an opportunity for countries with limited fiscal capacity to prioritise vulnerable subsets of the population.

Kenya is using SHI as the mechanism for working towards UHC and is in the process of implementing comprehensive reforms to revamp the existing health insurance framework.^19 20^ In October 2023, the government of Kenya launched a national UHC strategy known as ‘afya nyumbani’ (translated as ‘health in the household’), emphasising the significance of primary healthcare in ensuring healthcare accessibility for all.^21^ As a part of this strategy, the government has enacted pivotal health laws to establish the necessary legal framework for achieving UHC.^22 23^ Among the key goals of these legal reforms is the establishment of an SHI fund anchored within the SHI Act of 2023 and the move to mandate membership into the fund for all Kenyan citizens and residents. According to this Act, the SHI fund will receive funding from both government tax budgetary allocations and individual household contributions. Contributions from Kenyans in formal employment will be collected through pro-rata direct payroll deductions, while a proxy-means testing process will be employed to identify those in need, granting them premium exemptions and determining contributions for households in the informal sector. The Act also specifies that indigent households will be covered through contributions from the government tax budget allocation. All beneficiaries under the SHI scheme will be assured access to an essential benefit package.

Population coverage through SHI remains low and highly inequitable in LMICs.^24^ In systematic analysis of 29 SHI schemes in LMICs, it was found that the wealthiest individuals were 61% more likely to enrol compared with poor households.^25^ To overcome the challenge of including poor households, many countries are turning to premium exemptions or subsidies for the poor financed through public and donor funds on behalf of the poor.^26^ Ghana and Rwanda have premium exemption policies for indigents and vulnerable populations.^5 27 28^ Other countries such as Peru, Mexico, Nepal and Burkina Faso subsidise premium contributions for poor households.^29 30^ Despite best efforts by countries to target vulnerable households through various methods, these efforts have been largely unsuccessful in ensuring the equitable inclusion of poor households in SHI.^29^

Prior attempts to cover indigent households in Kenya exist. In 2014, the Government of Kenya launched a National Health Insurance Subsidy Programme (HISP) under the national insurer, the National Health Insurance Fund (NHIF) initially targeting poor orphans and vulnerable children in the pilot phase.^31^ Households included in the programme were identified from the national poverty list maintained by the ministry of labour and social services. Subsequent scale up of the programme in 2016 targeted more households with the intention of progressively covering the poorest 10% of the population. Evidence, however, suggests that the HISP programme did not reach the intended beneficiaries.^32^ An assessment of the HISP programme found that 65% of the beneficiaries belonged to the wealthiest socioeconomic quintiles.^31^ Other notable attempts to cover poor households followed the national government-driven UHC pilots in the counties of Kisumu, Nyeri, Isiolo and Machakos in 2018. Residents of these counties were enrolled in a UHC cover referred to as ‘afyacare’.^33^ In what was considered the UHC scale up, the national government in 2021 targeted to provide 1 million poor (indigent) households with a comprehensive NHIF cover.^34 35^

Gender roles and societal norms often translate to women having lower incomes and limited control over household finances compared with men in LMICs.^36^ This makes it particularly difficult for women to afford out-of-pocket (OOP) healthcare costs, forcing them to make difficult choices between healthcare and other essential needs like food and education. In addition, the burden of OOP payments can exacerbate existing gender inequalities in health outcomes.^37^ When women are unable to afford necessary healthcare, it not only impacts their own health but can also have long-term consequences for their families and communities.

The design and implementation of targeting programmes globally is riddled with numerous challenges including inadequate funding and capacity gaps.^35^ These challenges lead to inclusion of non-poor households (inclusion errors) and exclusion of true poor households (exclusion errors) in and from subsidy programmes.^35^ Evidence shows that accuracy of beneficiary identification is both a function of the targeting methods used as well as the skills and capacity of the various actors involved in the implementation process.^38^ A variety of beneficiary targeting strategies have been documented and they fall into two main broad categories: direct and indirect targeting. Direct targeting involves employing means and proxy tests that use a set of household expenditure data and household characteristics strongly correlated with income or wealth, such as household size, education level and ownership of assets, to estimate household income, respectively. The advantage of these methods lies in their accuracy and verifiability, achieved through observable household indicators like living conditions (permanent house, roofing type, etc). However, these methods are often costly and administratively burdensome, limiting their use in LMICs despite their precision.^39 40^

Indirect targeting adopts less rigorous means, incorporating SES (formal or informal occupation), demographics (gender and age), and geographical location as criteria for identifying households eligible for subsidies .^26^ Kenya has used a combination of both in the past to include the poor households identified through direct targeting while indirect targeting has been employed to include vulnerable population subsets like pregnant women, people living with disabilities and the elderly.^41^ Studies from different countries have also documented different actors charged with the identification of indigent households including local government, municipalities and authorities, ministries of health, primary care units, departments of social protection.^30^

Ultimately, a robust subsidy programme is one that accurately identifies beneficiaries and provides financial risk protection to enrolled households. Disaggregated data on catastrophic health expenditure (CHE) suggest that the poor are disproportionately affected by CHE. However, there is limited evidence on the effectiveness of health insurance subsidies in reducing healthcare costs and improving utilisation among poor households.^26 42^As Kenya rolls out the recently unveiled UHC strategy that includes a national indigent cover programme, the goal of this study is to evaluate the impact of health insurance subsidy on poor households’ healthcare costs and utilisation. We will also assess the effectiveness and equity of the beneficiary identification approach employed. This study is part of a broader evaluation of the national UHC indigent programme with the qualitative components forming a separate but related study.

## Study objectives

### Overarching objective

The overall aim of this study is to assess the impact of the National Health Insurance Subsidy Programme under the national UHC programme.

### Specific objectives

#### Specific objective 1

Assess the impact of the indigent programme on financial risk protection and healthcare utilisation among indigent households.

#### Specific objective 2

Determine the effectiveness and equity of beneficiary identification mechanisms in the UHC indigent programme.

#### Specific objective 3

Determine the gendered effect of the UHC indigent programme.

## Study justification

As Kenya rolls out its UHC plans under the national SHI fund, it is crucial to assess the impact of the health insurance subsidy on household health expenditure among indigent households. Specifically, this study will provide evidence on the extent of financial protection by comparing OOP healthcare costs between indigent households with the insurance cover and those without. This evidence is important to inform the health benefit package purchased on behalf of poor households at different levels of the health system. In addition, the effectiveness of the beneficiary identification under the UHC strategy will provide evidence informing the strengthening of the subsidy programme to improve its impact and equity.

In addition, designing and implementing UHC indigent programmes that are responsive to the gendered nature of OOP healthcare burdens is crucial in LMICs.^37^ This includes ensuring that programmes are designed with an understanding of the specific healthcare needs of women and addressing the financial barriers they face in accessing services. By analysing the impact of UHC indigent programmes on OOP payments by gender, our research can will provide critical evidence for policymakers and programme implementers working to achieve health equity in LMICs. As many LMICs turn to SHI to provide population coverage, lessons from the implementation of SHI with a subsidy programme in Kenya will provide key lessons for other countries. Collectively, this body of work contributes to global evidence on UHC implementation strategies that work in LMICs.

## Methods and analysis

### Theory of change

The study theory of change is summarised in table 1 outlining the reform inputs, activities, outputs, intermediate outcomes and the envisioned long-term impact. The basis of the national indigent programme rests on strategic reforms in policy and legal frameworks, establishment of clear implementation guidelines, budget allocation for subsidies, definition of a comprehensive benefit package and the development of means testing tools.^22 23 38 43^

**Table 1:** Theory of change

Reform input	Reform activities	Reform output	Intermediate outcomes	Long-term outcomes
Policy/legal framework Implementation guidelines Budget allocation for subsidies Defined benefit package Means testing tools	Identification of beneficiaries Registration of beneficiaries Disbursement of allocated funds to NSHIF Service utilisation by beneficiaries Sensitisation of beneficiaries Beneficiary feedback	Eligible beneficiary database Registered beneficiaries Funds held at NSHIF Sensitised beneficiaries	Increased number of poor/vulnerable covered by NSHIF Reduced OOP among the poor/vulnerable Reduced incidence of CHE Increased service utilisation among the poor/vulnerable	Improved equity in financing Improved equity in service utilisation Improved health outcomes

Constraints	Unintended effects
Inadequate funds allocated to the programme Funding flows constraints (delays in disbursements to NSHIF) Difficulties in identifying beneficiaries. Political interference	Inclusion and exclusion errors in beneficiary identification Excluded households unable to access healthcare

CHEcatastrophic health expenditureNSHIFNational Social Health Insurance FundOOPout of pockect

Many policy activities are expected to follow the initiated reforms. This involves the identification and registration of beneficiaries, seamless disbursement of allocated funds to the National Social Health Insurance Fund (NSHIF) and active engagement in service utilisation by the beneficiaries.^22 26^ Furthermore, a key aspect of this phase is the sensitisation of beneficiaries, ensuring they are well informed about their entitlements and encouraging their active participation.^5 44^ Continuous feedback mechanisms will need to be established to capture the perspectives and experiences of the beneficiaries.

As a result of these activities, tangible outputs are expected to be realised, including a robust and accurate eligible beneficiary database, a comprehensive registry of registered beneficiaries and funds securely held at the NSHIF.^45^ Additionally, beneficiaries are sensitised to their membership and their entitlements. The successful implementation of these reform activities is expected to consequently yield intermediate outcomes. The programme achieves an increased number of poor and vulnerable individuals covered by the NSHIF. The incidence of OOP among the targeted demographic is reduced, thereby alleviating financial burdens of seeking healthcare.^29^ Moreover, the incidence and intensity of CHE are expected to diminish while service utilisation among the poor and vulnerable is expected to increase.^46^

The overarching goal of the policy reforms is the realisation of long-term outcomes. With increased coverage among the poor and vulnerable, it is expected that there will be significant improvement in equity in financing, and enhancement in equitable utilisation of health services. These outcomes culminate in improved health outcomes, underscoring the programme’s effectiveness in not only facilitating utilisation of healthcare services but also in positively impacting the health status of the covered population.^47 48^

We are mindful of potential constraints and unintended effects; adequacy of funds allocated to the programme, potential delays in funding flows to the NSHIF, challenges in identifying beneficiaries and instances of political interference are identified as potential constraints.^26 30 49 50^ Additionally, there is a recognition of the possibility of inclusion and exclusion errors during the beneficiary identification process which may lead to excluded poor households not accessing care.^35^

### The intervention

The plan to cover vulnerable households is extensively mentioned in the ongoing legal reforms that aim to transform healthcare services accessibility and the financing mechanisms for these services. The SHI Act 2023 describes an indigent as ‘a person who is poor and needy to the extent that the person cannot meet their basic necessities of life’. It further specifies the eligibility into the indigent programme that will be determined through means testing administered in a household assessment to determine the SES. The Kenyan government has committed to paying annual health insurance premiums on behalf of households lacking the ability to pay. These premiums will be financed through government budget allocation channelled through the NSHIF.^22^ The SHI is expected to cover a standard benefit package in secondary and tertiary thereby guaranteeing indigents access to a standard package similar to contributing beneficiaries.

#### Study setting

We will purposively select counties representing varying geographical contexts and varying progress with the rollout of the UHC indigent programme based on existing reports from Ministry of Health (MoH) and county of government. Based on these criteria, counties are likely to include Kisumu, Kilifi and Kiambu. Table 2 shows the county profiles.

**Table 2:** Study county profiles

	Kisumu	Kilifi	Kiambu	National
Population 2019 census (n)	1 155 574	1 453 787	2 417 735	47 564 296
Poverty rates (%)	36.3	49.2	20.5	38.6
Health insurance coverage (%)	18.1	11.8	39.1	26.3
Average household size (n)	3.8	4.8	3.0	3.9
Criteria considered in selection	One of the four UHC pilot counties	High poverty rates. Coastal region	Relatively higher SES and periurban County	

Source: References 59 60 68.

SESsocioeconomic statusUHCuniversal health coverage

### Overall study design

This study will employ quantitative study design with both quasi-experimental and cross-sectional study designs. A quasi-experimental cohort study design will be used to evaluate the impact of the indigent programme on households’ expenditure and health service utilisation while a cross-sectional study design will be used to assess the effectiveness and equity in the beneficiary targeting mechanisms.

#### Prospective cohort study: impact evaluation

##### Study design

The first specific objective of this study will employ a prospective matched cohort design with households matched using characteristics that include household size, SES (asset index) and county of residence. The cohort will recruit households to an ‘exposure’ arm and a control arm and follow these households for 12 months, collecting data in four survey waves in 3 months intervals. The ‘exposure’’ arm will comprise households that have been enrolled on the UHC indigent programme. In contrast, the control arm will comprise households that meet the eligibility criteria for enrolment in the UHC programme but have not been enrolled. The primary outcome will be the proportion of OOP measured against household expenditure.

Inclusion criteria:

The inclusion criteria of this study will be:

1. Poor or vulnerable households that meet the eligibility criteria for enrolment in the UHC indigent programme.
2. Household respondents who are at least 18 years and above and have adequate knowledge of households spending and health-seeking events.
3. Availability for a 12-month follow-up period.

Specific objective 2 will use a cross-sectional study design to assess the effectiveness of the poverty targeting mechanism employed in the UHC indigent programme by analysing existing household data collected in the first wave/baseline of households’ survey from households in the exposure arm in specific objective 1 above. The primary outcome will be the level of inclusion errors (table 3) with the households forming the unit of analysis.

**Table 3:** Inclusion and exclusion errors

	Welfare status of households	
	Poor	Non-poor
**Households excluded from programme**	Exclusion error(under coverage)	Successful exclusion
**Households included in programme**	Successful targeting	Inclusion error(leakage)

Source: Coady and Hoddinott.^69^

Specific objective 3: To include a gender equity evaluation in the study, conduct subgroup analyses within each gender to assess (1) how the UHC indigent programme’s impact on the level of OOP costs and CHE differs between men-led and women-led households as well as the household healthcare costs disaggregated by gender of household members (men vs women) and (2) the enrolment into the indigent programme across the genders. We will, therefore, investigate whether there are gender-based differences in enrolment and utilisation of the UHC indigent programme, and how this may influence the primary outcome.

Table 4 indicates the study methods to be employed per objective.

**Table 4:** Data collection methods per objective

	Specific objective	Sampling	Methods	Variables	Outcome	Analysis
1	To assess the impact of the indigent programme on financial risk protection and healthcare utilisation among indigent householdsWhat is the impact of SHI premiums subsidy on household healthcare utilisation and healthcare cost?	Purposive-county levelHH sampling- Stratified random sampling.n=675 per armTotal 1350	A prospective matched cohort design with a household survey	Medical cost-outpatient, routine, InpatientNon-medical—transport to facilityWeekly, monthly and annual food and non-food household expenditure and household assetsHH characteristics: gender of the household head, household size, education level, health insurance status, perceived health status, marital status.	CHE—using the 40% threshold OOP payments (outpatient and inpatient costs) Depth of cover Likelihood of incurring CHE	Descriptives Incidence and intensity of CHE: CHE headcount and overshoot Impoverishment (being pushed further into poverty) Conditional regression
2	To determine the effectiveness and equity of beneficiary identification mechanisms in the UHC indigent programmeHow effective and equitable is the beneficiary identification programme?	Secondary analysis of baseline data of households in the exposure armn=675	A cross-sectional study design	Household assetsHH characteristics: gender of the household head, household size, education level, health insurance status, perceived health status, marital status.	Inclusion errors	DescriptivesCorelation
3	To determine the gendered effect of the UHC indigent programmeTo what extent does the indigent programme contribute to bridging gender disparities in healthcare utilisation among the poor?	Stratified random sampling.n=675 per armTotal 1350Secondary analysis of baseline data of households in the exposure arm	Prospective matched cohort design-gendered experience of CHECross sectional study design-gendered selection of beneficiaries	Medical cost-outpatient, routine, InpatientNon-medical—transport to facilityWeekly, monthly and annual food and non-food household expenditure and household assetsHH characteristics: gender of the household head, household size, education level, health insurance status, perceived health status, marital status.	Proportion of women-led led included in subsidy programmeHealthcare utilisation by genderOOP costs across by gender of household membersCHE by gender	Proportion by gender, χ^2^ ORs

CHEcatastrophic health expenditureHHHouseholdOOPout of pocketSHISocial Health InsuranceUHCuniversal health coverage

##### Study population

The study population will be all households eligible for the national UHC indigent programme.

##### Setting

Kenya is a middle-income country with a population size of 49 million people and a Gross Domestic Product (GDP) per capita of 2219.^51 52^ The country runs a devolved government system with a national government and 47 county governments.^53^ The national government is charged with policy formulation and health service provision at national referral hospitals while county governments oversee primary and secondary healthcare provision within the counties. There are six levels of the health system namely community, dispensaries, health centres, subcounty hospital, county referral hospitals and the national referral hospitals.^54 55^ The health sector is financed through taxes, external funds and private contributions by households in the form of health insurance and OOP payment.^56^ Health services are provided by both public and private health facilities.

Over 70% of the Kenyan population live in rural settings and the informal sector constitutes 83% of the workforce in the country.^57 58^ Poverty rates within the country are estimated at nearly 40% of Kenyans.^59^ According to the recent Kenya Demographic Health survey, 26% of the population is covered by health insurance.^60^ Approximately 7.1% of Kenyan households incur CHE and up to 1 million Kenyans are pushed into poverty every year because of OOP payments.^42^

##### Sample size and sampling

Multilevel sampling will be employed in determining households to include in the study. In the first stage, 3 counties will be purposively sampled from the 47 counties in Kenya. The selection will be informed by the desire for geographical diversity and variability of related indicators such as population of the county, households’ sizes and poverty levels. We will also consider levels of progress recorded by the MOH in implementing the UHC indigent programme in the three counties. The proposed counties are Kisumu, Kilifi and Kiambu.

We estimated that a minimum sample of 179 households per comparison group would have 80% power to detect a 15 percentage point difference in the proportion of OOP healthcare costs as a share of total household expenditure, assuming a proportion of OOP costs of 40% in the control group, a design effect (DE) of 1.2 and a two-sided alpha level of 0.05.

The initial sample size was calculated using the following formula for comparing two proportions^61^:

$$n=\frac{{\left(Z_{\alpha /2}+Z_{\beta }\right)}^{2}\left[p_{1}\left(1-p_{1}\right)+p_{2}\left(1-p_{2}\right)\right]}{{\left(p_{1}-p_{2}\right)}^{2}}$$

Where:

- n=required sample size for each comparison group.
- Z_α/2_=Z-score for the desired alpha level (0.05 for a two-sided test corresponds to 1.96).
- Z_β_ = Z-score for power (0.80 for 80% power corresponds to 0.84).
- p_1_=Estimated proportion of the outcome in the control group (40%=0.440).
- p_2_=0.55 (proportion in the intervention group, reflecting a 15 percentage point difference).
- p_1_−p_2_=Expected difference in proportions (15 percentage points=0.15).
- DEFF=design effect (1.2).

The DE of 1.2 was chosen based on typical values reported in the literature for health-related household surveys where clustering occurs. In studies with moderate clustering (such as those based on households within geographical regions or communities), a DE between 1.1 and 1.5 is often observed.^62^ Using these parameters, the base sample size was estimated to be 179 households per group. Given that up to 60% of the study participants may be lost to follow-up due to refusals or the nature of the conditions they live with over 1 year, the sample size was adjusted by multiplying the initial estimate by 1/0.4 to account for a 40% retention rate. Additionally, to allow for oversampling, the sample size was further increased by 50%. The final sample size per group is calculated as:

n=179×1/0.4×1.5.

n=671 households per group.

To ensure a more practical sample size, we rounded the final number to 675 households per group, resulting in a total of 1350 households across both study arms.^63^

In the second stage, we will employ stratified random sampling using counties as the stratum. The sample size will be uniformly distributed across the three counties with equal proportions for urban and rural populations.

The sampling frame will be provided by the national list of indigents identified for enrolment in the national indigent programme. The sampling frame will then be grouped into the households enrolled in the national programme as the exposure group and eligible households not enrolled in the UHC programme as the control group. An initial consultation with inform the source of the indigent list to be used as the sampling frame in this study.

###### Subspecific objective 2

For the assessment of the effectiveness of the targeting programme, we will include all the households in the exposure arm of the study.

### Data collection

Household survey data will be collected through an interviewer-administered structured questionnaire within households. Interviewers will make three contact attempts as a baseline to reach the selected household respondent, with the possibility of extending these attempts in the initial waves to maximise response rates. Contact times and days will be varied to increase the likelihood of reaching households. Clear communication will be provided to explain the study’s importance and data confidentiality to minimise refusal.

Households that remain unavailable for interviews after the final attempt or refuse participation will be classified as non-responses for that wave. However, non-respondent households from earlier waves will be reapproached in subsequent waves to reduce potential bias and account for changes in household availability. The same contact protocol used in the initial wave will be consistently applied in the later waves. Reasons for non-response will be tracked, and the characteristics of respondents and non-respondents will be compared across waves to assess potential biases.

The questions included in the households’ survey questionnaire are adopted from the Kenya Health Expenditure and Utilisation survey. The English questionnaire will be translated into the national language (Kiswahili) version to provide households with preferred language options for responding. The questionnaires will be administered to the selected households by data collectors. Each household interview is estimated to take between 40 and 60 min per household.

A team of interviewers with experience in conducting national surveys will be identified with the assistance of the respective county leadership and supervisors will be drawn from the MoH and other government ministries as well as county staff. The interviewers will undergo training on data collection using tablet-based questionnaires and on survey concepts. They will also be provided with printed consent forms and study manuals containing study procedures and guidelines.

All data collectors will be required to maintain field notebooks to document their general data collection experiences. These experiences will be shared during supervision and debriefing sessions. For the matched cohort study, data collection will be done in four waves at 3-month intervals. We will collect household demographics, health-seeking events and costs and the household expenses as well as ownership of assets. Healthcare costs will be recorded for outpatient, inpatient and routine healthcare visits. We will collect both direct medical costs (consultation, medicines and diagnostics) as well as direct non-medical costs such as transport related to health-seeking episodes in the household. Weekly, monthly and annual food and non-food household expenditure and household assets will be collected to assess households’ SES. Other variables of interest will include household size, gender of household head, education level, health insurance status, perceived health status, marital status, income level and asset wealth.

### Data analysis

To determine the difference between the ‘exposure’ and control group, we will employ the coarsened exact matching method to pair households in the two arms (control and exposure) based on their baseline characteristics. The variables used to match these households include^1^ household size,^2^ county of residence and^3^ household SES.

The primary outcome of interest in this study is the ‘proportion of OOP’. This outcome measures the proportion of households in each arm that experience CHE. We will, therefore, estimate the OOP payments for the exposure and control group. To calculate CHE, we will express total household OOP as a share of total non-food household expenditure over 1 year. We defined CHE as when OOP exceeds 40% of non-food expenditure as established in the literature.^31^ The incidence of CHE is represented by the proportion of households that incur CHE.

We will compute means for costs that are reimbursed by the NSHIF and those covered using OOP payments. The study will employ Pearson’s χ^2^, Kruskal-Wallis and Mann-Whitney tests to examine differences in OOP expenses as a proportion of total annual household expenditure and the incidence of incurring CHE. We will also estimate the CHE and fit a conditional regression model to assess the likelihood of incurring CHE among the two arms. The choice of covariates to be included in the model will be guided by existing literature to account for potential confounding factors or variables that may influence the OOP. These may include socioeconomic, demographic and health-related variables like income and level of education, age, gender, health utilisation and status among others.^42 64 65^

To assess the effectiveness and equity in beneficiary identification, we will estimate the inclusion errors of the targeting programme. Our interest will be to determine the proportion of non-poor households identified as poor and enrolled into the programme otherwise known as leakage.

We will use principal component analysis to estimate household SES scores of the households enrolled in the indigent programme. To assess the socioeconomic characteristics of households enrolled in the indigent programme, we will use principal component analysis (PCA). PCA is a statistical method that reduces the dimensionality of data by identifying a smaller set of uncorrelated variables, called principal components, that capture most of the variance in the original variables.^66^ In our analysis, we will consider a range of variables relevant to household SES, including (1) type of dwelling (eg, owned, rented, shared), the number of rooms and access to basic amenities (eg, electricity, sanitation) and (2) ownership of durable goods such as televisions, refrigerators and bicycles. This variable list is derived from the Kenya Health Equity tool,^67^ which was developed for demographical health surveys and validated for Kenya. The first principal component derived from this analysis will represent a composite measure of household SES, with higher scores indicating higher SES in our sample. We will then map these scores against the asset score boundaries of SES quintiles of the Kenya demographic health survey 2022. The proportion of the households enrolled in the indigent programme that belongs to Q3–Q5 in the national socioeconomic quintiles will constitute the inclusion errors. In addition, we will disaggregate these data based on gender of the household head to determine the gender equity in the households covered by the indigent programme.

In this study, we aim to explore the gendered impact of the programme on household health expenditure. We define ‘men-led’ and ‘women-led’ households based on the self-reported gender of the household head. The household survey includes questions that ascertain the roles of household members, enabling us to distinguish between different household leadership structures accurately.

### Validity

To ensure validity in this study, the data collection tools will be pretested in pilot households within the study area, not included in the study sample, to identify and rectify potential issues and assess the interview duration. We will ensure thorough training of study enumerators to ensure reliability.

To ensure content validity, we will define and adopt standard measures the primary outcome, ‘incidence of CHE’, in our study protocol and specify how it will be measured. Our measurement tools, including survey questions and data collection instruments, will be selected or developed to align with the construct of CHE.

Content validation will be ensured by inviting experts in the field of healthcare economics or health policy to review the study design, sampling strategy and the measurement tools we plan to use. Their feedback and recommendations will be integrated to enhance the study’s validity.

For sampling validity, the eligibility criteria for both the ‘exposure’ and ‘control’ arms will be defined and adhered to meticulously to minimise selection bias. Quality control measures including adequate training of data collectors, data validation checks as well as regular audits during data collection process will be ensured to minimise data entry errors, reduce missing data and ensure the proper handling of data. We will also use appropriate statistical methods to analyse the data, ensuring that our analysis aligns with the study objectives and the validity of our measurement tools. Lastly, quantitative data resulting from this study will be triangulated with qualitative data from a broader study.

### Pilot study

A pilot study will be carried out to evaluate the research protocols, data collection instruments and sampling methods ahead of the primary study. This pilot will take place in Kiambu County, where 50 households will be selected, with half of them being enrolled in the indigent programme and the other half being eligible for the programme but not enrolled. The pilot county is chosen because it is one of the purposively selected counties for data collection and presents the most logistically feasible county of the three. The feedback and findings from the pilot will guide enhancements to the data collection tools, protocols and data collection procedures.

### Data management

The household survey data collected via RedCap will be securely stored on Kenya Medical Research Institute (KEMRI)-Wellcome Trust servers in accordance with KEMRI-Wellcome Trust ICT and Data Management policies. To ensure data quality and completeness, appropriate skip patterns as well as range and completeness checks will be included in the tool. A thorough data cleaning process will also be conducted before the commencement of analysis. Data will be deidentified to protect the confidentiality of household information. Deidentified data files will be securely shared with the research team through a protected file transfer format and maintained on password-protected devices.

## Ethical consideration and dissemination

Before commencing the study, approval was sought from the Scientific and Ethics Review Unit at KEMRI who will review it for scientific and ethical considerations (online supplemental file 1). The respective County Health Department will also grant additional permission for conducting the studies in their counties. Participants who agree to participate in the study will provide written informed consent for the interviews. Dissemination strategies include peer-reviewed publications, conference presentations and policy-maker engagement for broad accessibility and impact.

## Population involvement

Collaborative discussions have provided valuable perspectives on the impact of health insurance subsidies on households’ healthcare experiences and have contributed to the development of research questions that resonate with the concerns of the study population. Household representatives will be recruited to provide insight into the household-level impact of the indigent programme.

## Study challenges and limitations

The primary concern in this study is dynamic nature of UHC reforms on the implementation of the intervention. As a result, the intervention described here, along with its implementation, is constantly evolving including the implementation start date. In addition, during the implementation of the indigent programme, obtaining a reliable and verified list of indigent households may prove challenging due to uncertainties regarding which institution will act as the custodian of the indigent list database. This could impact the process of identifying and enrolling study participants. To address this, we will explore various potential sources for the indigent list, including the county department of health, the SHI fund, the community health assessment and the ministry of labour and social services. It is likely that triangulating these sources will be necessary to develop a comprehensive and reliable database for recruiting study participants.

Another challenge is related to the longitudinal nature of the study design. Since the study spans 12 months with multiple data collection points, there is a risk of significant loss to follow-up. Some participants may move, drop out or become unavailable, which can affect the study’s internal validity and generalisability of results. To mitigate this, the dropout rate has been factored into the sampling procedure with provisions based on households’ studies carried out in similar settings.

Lastly, collecting accurate data on household health expenditure can be challenging. Respondents may provide inaccurate or incomplete information, leading to measurement error. In addition, given the gender focus of our study, we recognise the potential for recall bias, especially where male household heads may not be fully aware of healthcare spending for female household members. To address this, enumerators will be trained to probe for accurate household health expenditure and, where possible, involve the household member most knowledgeable about healthcare costs in the response process. In most cases, household survey data suggest that women tend to be more familiar with healthcare spending.

Nonetheless, a few assumptions, however, must hold for this study. First, poverty and vulnerability assessment will have to be done in a cross-sectional to a large subset of the population. Second is that following the poverty assessment, there needs to be a phased rollout of the indigent programme will allow for the assessment of households included in the first phase compared with the households who are eligible but scheduled to be enrolled in subsequent phases. Third is that the period between the phases will be at least 12 months to allow for the follow-up period proposed in our prospective study. Regular monitoring and adaptation of study procedures may be necessary to address these challenges as they arise. In the absence of these conditions, we will explore a secondary design that will reflect the prevailing rollout plan at the time and provide evidence of the impact, effectiveness and equity of the indigent programme and the beneficiary identification process.

## supplementary material

10.1136/bmjopen-2024-083971 online supplemental file 1
