# Supplementary material for: Evaluating the effectiveness of the National Health Insurance Subsidy Programme within Kenya’s universal health coverage initiative: a study protocol
Source: BMJ Open. 2024 Nov 21;14(11):e083971. doi: 10.1136/bmjopen-2024-083971 (PMC11590815; doi:10.1136/bmjopen-2024-083971)
Supplement: Supplementary file 2 [file bmjopen-2024-083971.reviewer_comments.pdf]

PEER REVIEW HISTORY

BMJ Open publishes all reviews undertaken for accepted manuscripts. Reviewers are asked to complete a checklist review form and are provided with free text boxes to elaborate on their assessment. These free text comments are reproduced below.

ARTICLE DETAILS

Title (Provisional)

Evaluating the Effectiveness of the National Health Insurance Subsidy Programme within Kenya's Universal Health Coverage Initiative: A Study Protocol

Authors

Maritim, Beryl; Nzinga, Jacinta; Tsofa, Benjamin; Musiega, Anita; Mugo, Peter Mwangi; Wong, Ethan; Mazzilli, Caitlin; Ng'an'ga, Wangari; Hagedorn, Brittany; Turner, Gillian; Musuva, Anne; Murira, Felix; Ravishankar, Nirmala; Barasa, Edwine

---

VERSION 1 - REVIEW

**Reviewer**	**1**
**Name**	**Wagner, Adam**
**Affiliation**	**NIHR Applied Research Collaboration (ARC) East of England (EoE)**
**Date**	**11-Feb-2024**
**COI**	**None**

Thank you for the opportunity to review this article that addresses an important area. I think the analysis plan is broadly reasonable and sensible, but clarity is needed around the primary outcome (this becomes clearer, but initially, it fluctuates be CHE and OOP – the link between these comes explicitly relatively late in the manuscript) and presentation of the associated sample size calculation. Also, the motivation for exploration around gender needs to be provided, and some further detail/clarity on how this will be explored/analysed. These two general issues led to me selecting "No" for the Review Checklist, for items 4 ("4. Are the methods described sufficiently to allow the study to be repeated?") and 6 ("6. Are the outcomes clearly defined?")

My feedback was primarily written linearly – in parts, you may provide detail later in your manuscript that address my points – where this is the case, I would encourage you to signpost to the later sections.

I structure my feedback in decreasing importance – the main substantive issues first, then subsequently to less important issues like typos etc.

Main issues:

- My main point of concern is around sub-objective 3 – the gendered impact of the program – its motivation and detail of its analyses. First, this sub-objective is not motivated in the

background, nor in the study justification. Next, there is reference made to “expenditure differs between male and female led households” – how is this going to be defined/ascertained? Are there any concerns about data collectors of one gender being more likely to consider households as being led by that gender? Also, when this sub-objective is primarily motivated as sub-group analysis, why does it focus on CHE, when sub-objective 1 focuses on OOPs? Will the analysis take account of the gender of the household representatives responding? (Might men representatives be less familiar with the health spending of women in the household?)
(Please also distinguish sex and gender – in this setting, I would consider ‘gender’ most appropriate, but, in line with that, language should align with men and women, rather than males and females).

- In line with the above point, table 6 does not address how households are going to be allocated by gender – for example for “CHE by gender” – there is no mention of the household lead gender among household characteristics. Also, more detail needs to be given on how healthcare utilisation and OOP costs by gender are going to analysed – will this still be at the level of household (so, based on household lead gender?), or will the unit of analysis change to individual from household (how will this take account of clustering? E.g. people living in the same household). Or, might percentage of total spending on one gender be considered?
- There is inconsistency in what the “primary outcome” is: in section 4.3.1.1, we have “primary outcome will be the proportion of OOP.”, while the sample size calculation is based on chronic health expenditure – these must be consistent. One would expect the sample size calculation to be based on the primary outcome, so should section 4.3.1.1 make reference to CHE? Relatedly, Table 4 could be more explicit in referring to the primary outcome for sub-objective 1. Even within the sample size calculation, there is confusion as to the primary outcome: p16, L13-15: “(e.g., proportion of households incurring catastrophic health expenditure)”; L51-56: “This calculation is based on an assumed out-of- pocket cost proportion of 13% in the control group (based on KHHUES 2018 incidence of CHE in Q1)”. This becomes clearer with: P20, L22-25: “The primary outcome of interest in this study is the "proportion of OOP." This outcome measures the proportion of households in each arm that experience catastrophic health expenditure.” – this is the first time that OOP and CHE have been related – this needs to be done much earlier to alleviate the confusion around the definition of primary outcome.

- More care needs to be taken in the presentation of the sample size calculation on P16. Please address the following:
    - Provide a reference for where you have taken this formula from
    - Be careful in the formatting: I suspect the “+” before Z_beta should not be subscript.
    - In the explanation of formula terms, a number of text elements need to be made subscript (e.g. $\alpha/2$). Also, “(13.1% = 0.)” and “*(8 percentage points = 0.08)*” should be corrected, and made consistent. “*p = Estimated proportion of the outcome*” – the “p” needs a subscript.
    - The bullet point for effect size specifies it as 1.2 (“DEFF = Design effect (1.2)”), while the text has this as 2 (“a design effect of 2”).
    - Why has a design effect of 1.2 been chosen? Some justification is needed.
- P21, L10-13: “We will use principal component analysis to estimate household SES scores of the households enrolled in the indigent programme.” – please provide more detail of this approach – for example, what variables will be considered in the PCA? You later comment: “In addition, we will disaggregate these data based on gender to determine the gender equity in the households covered by the indigent programme.” – again, detail is needed on how you will categorise gender – gender of the household lead?

Minor:

- Strength and limitations: The first three statements are statements of methods – they don't highlight to the reader why they are strengths – why are they better than some alternative methods? Why should the reader consider them strengths?
- P5, L21/22: "Kenya is utilizing SHI as the mechanism for working towards UHC" – can you give examples of other countries where such a transition has worked/adopted this approach?
- Specific objective 1: Previously, language has focused on 'utilisation', here you switch to 'access' – is there a reason for this? I would consider 'utilisation' the broader term, and encourage you to stick with this, unless you can justify a reason not to. Also, you talk about 'financial risk protection' – would it be useful to be more specific and specify 'catastrophic health expenditure' in some fashion?
- Specific objective 3 is not motivated in the background – nor is it touched on in the Study justification.
- P12, L21-26: "The SHI is expected to cover a standard benefit package in secondary and tertiary thereby guarantee indigents access to a standard package similar to contributing beneficiaries."- I'm somewhat unclear if the 'standard package' will be 'running' during the duration of this evaluation – will we know what contributing beneficiaries receive?
- P13, L33-36: "The primary outcome will be the proportion of OOP." – please specify the denominator – household income?
- P15, L40-42: "Poverty rates within the country are estimated that nearly 40% of Kenyans [54]." – I do not believe the 'poverty' has been defined in the Kenyan setting – how is this operationalised?
- Is Table 5 really required?
- Table 6:
  - Again, please be consistent with labelling – are these objectives, or specific objectives etc
  - "CHE-40%" – I don't understand what the "40%" means here [Resolved later, but no link between explicit between OOP and CHE until later]
  - In outcomes for objectives 3, "Proportion of women included in subsidy programme" – I don't know precisely what this means – proportion of households that include women?
- P19, L10-15: "Households that are not available for interviewing, either due to absence on the third attempt or a refusal, will be excluded from the survey and considered as non-responses." How does this relate to waves of the study? (e.g. "collecting data in four survey waves in 3 months intervals."). If the household is defined as non-response in wave 1, will they be approached during the subsequent waves?
- P19, L19-26: Will there be any piloting of the translated questionnaire? From later reading, I see this is addressed in Section 4.3.2 – it may be useful to signpost the reader to this section.
- P20, L5-9: "Other variables of interest will include household size, education level, health insurance status, perceived health status, marital status, income level, asset wealth," – given the importance for sub-objective 3, it seems odd not to mention the lead status of the household – whether the household is led by a woman or a man. Also, note this extract needs to end with a full stop (currently ends in a comma).
- As I read BMJ Open guidance, I believe expected dates of the investigation should be provided – I don't believe these are?

Very minor:

- P1, Author list: stray spaces after first names and before semi-colons for Brittany and Gillian
- P4, L43/44: Typo: " .In" -> ". In"
- P4, L57/58: "Targeted approach focuses" -> "Targeted approaches focus"
- P5, L58: "remain" -> "remains"
- P6, L5/6: "premiums exemption" -> "premium exemptions"
- P7, L15: "two main broad categories-direct and indirect targeting." – perhaps replace the en-dash with a colon, or use an em-dash with spaces either side of it.
- P8, Specific objectives: It reads as redundant to me to start each objective with "To" – I would remove. Also, heading 2.2 should be pluralised – there are more than one specific objectives.
- P9, L16: "The basis if the" – "if" -> "of"
- P11, L22-27: "We are mindful of potential constraints and unintended effects. Adequacy of funds allocated to the program, potential delays in funding flows to the NSHIF, challenges in identifying beneficiaries, and instances of political interference are identified as potential constraints[26,30,50,51]." – The second sentence feels orphaned – perhaps end the first current sentence with a colon and then use semi-colons for the list in the second sentence?
- Table 1: Perhaps clearest to the reader to explain acronyms in the caption/below the table to make the table self explanatory? The formatting of the headings is inconsistent: why is "output" not capitalised, but "Outcomes" etc is? Why is "Utilization" capitalised?
- P12, L3-8: I find this sentence hard to parse:
"The current health insurance reforms outline the plan to cover vulnerable households is extensively mentioned to in the ongoing legal reforms that aim to transform healthcare services accessibility and the financing mechanisms for these services." – please consider editing/splitting up to improve readability. Should 'outline' be 'outlining'?
- P12, L12: "It further reveals…" – 'reveals' feels odd wording in this context – perhaps 'defines' or 'specifies' etc?
- P12, L13/14: first use of SES – please spell out SES, as its first use
- P12, L17-22: "The Kenyan government has committed to paying annual health insurance premiums on behalf of households lacking the ability to pay and will be finance through government budget allocation channeled through the NSHIF [22]." – the second half of the sentence needs editing – the Kenyan government won't be financed etc - at minimum, remove the "be"? However, the sentence could do with refinement.
- P12, L33 and L35: Spell out MoH and COG at first use. MoH =?= Ministry of Health. CoG=?
- P13, L8: "while cross-sectional" -> "while a cross-sectional" – insert 'a'
- In Section 4.3.1.1, the authors switch to using "sub-objective', while the corresponding section is labelled "Specific objective" – I would use consistent language between the sections
- Table 4: Please be consistent with labelling – are these Objectives, sub-objectives, or specific objectives?
- P15, L26: "There are 6 levels" -> "There are six levels"
- P15, L34/35: "payment[61].Health" – insert space after full stop
- P16, "program in the 3 counties." -> "program in the three counties."

- Ref 19 – space needed before available: “30];Available”
- Ref 35: Can greater detail be provided? How would a general reader go about accessing this?

Reviewer **2**

Name **Sriram, Shyamkumar**

Affiliation **University of South Carolina, Department of Health Services Policy and Management**

Date **11-May-2024**

COI **I have no competing interests**

It is interesting area of research that is beneficial to the society at large. It is vital for low-income countries to have UHC to provide financial protection to the population who access healthcare both in the public and private sector hospitals.

Reviewer **3**

Name **Otieku, Evans**

Affiliation **University of Ghana, Institute of Statistical, Social and Economic Research**

Date **21-May-2024**

COI **None**

This protocol is feasible for achieving the intended objectives. Some sections require clarity to improve the methodological rigor.

Abstract

Lines 30 to 35. Be precise about how recent the UHC strategy was introduced in Kenya. Same comment in the background section where you mentioned “recent”.

Page 2, line 53. You could clarify that you intend to estimate inclusion error associated the scheme.

Background

1. Page 4 lines 51 and 52. “The Act also specifies that indigent households will be covered through contributions from the government tax budget allocation”. Does this not make non-indigent household with similar characteristics suitable control group?

2. The point about social protection programs not reaching intended beneficiaries were made at least three times. I feel it is repetition and could be merged. Example, page 5 lines 35, 54 to 58.

3. The two sentences on page 6 lines 53 to 60 contradicts each other. On one hand you say there is evidence showing the poor are disproportionately affected by CHE and the other hand, you say there is limited evidence on it.

Study Justification

.... "provide evidence of the extent of the financial protection offered by the cover on indigent household" as opposed to what/who?

Study design

How different is point 4.3 from point 4.3.1.1?

The authors mentioned the intention to use a prospective cohort design. Is not clear how the prospective design will be applied in a household survey. In my view, prospective cohort design is more applicable at point of care. How are the authors operationalising socio economic status as a matching criterion?

Consider deleting Table 4 because Table 6 contain more detailed information about sampling, design, study objectives, etc.

Study challenges and limitations

.... the source documents providing a list of indigents cannot be verified....

Where was the list document obtained from that it cannot be verified?

Recast the statement to emphasize that obtaining reliable list of beneficiaries from one source may be challenging, demanding that you consult other relevant stakeholder institutions which may take some time due to bureaucratic processes of some sort.

General comment

Careful edit is required to address some typo and grammatical errors. Examples:

Page 11 line 25. The cohort will recruit ....

Page 13 line 40. Poverty rates within the country are estimated that nearly 40% of Kenyans.

VERSION 1 - AUTHOR RESPONSE

Comment	Authors Response
**Reviewer 1**	
My main point of concern is around sub-objective 3 – the gendered impact of the program – its motivation and	Thank you for your insightful feedback. Below is how we plan to address the points raised:

detail of its analyses. First, this sub-objective is not motivated in the background, nor in the study justification. Next, there is reference made to "expenditure differs between male and female led households" – how is this going to be defined/ascertained? Are there any concerns about data collectors of one gender being more likely to consider households as being led by that gender? Also, when this subobjective is primarily motivated as sub-group analysis, why does it focus on CHE, when subobjective 1 focuses on OOPs? Will the analysis take account of the gender of the household representatives responding? (Might men representatives be less familiar with the health spending of women in the household?)<br>(Please also distinguish sex and gender – in this setting, I would consider 'gender' most appropriate, but, in line with that, language should align with men and women, rather than males and females).	Motivation for sub-objective 3: We have now included a more explicit justification for the gender analysis in the background and study rationale. This section emphasizes the importance of understanding gendered differences in financial burden, especially in low-resource settings where healthcare expenditure is known to vary across households led by different genders.<br><br>Defining men- and women-led households: We will define a household as "man-led" or "woman-led" based on the self-reported role of the household head. In the survey, we will ask specific questions to determine the gender of the household head, as well as questions about the roles of other household members to ensure accuracy. Additionally, the data collection process will mitigate any potential biases regarding gender assumptions by adequately training the enumerators on identifying the correct household leadership structure.<br><br>Gender of household respondents and recall bias: we acknowledge the concern about recall bias, particularly regarding male respondents potentially being less familiar with healthcare spending for female members. To address this, we will train enumerators to probe for accurate expenditure data and, whenever possible, request that the most knowledgeable household member on healthcare spending provides the responses. Furthermore, the survey design includes selecting respondents who are best positioned to answer questions on household spending.<br><br>Focus on catastrophic health expenditure (CHE): **The primary outcome of this sub-objective is proportion of out-of-pocket payments (OOP)**. This will also allow us to compute catastrophic health expenditure (CHE) based on the proportion of the household budget spent on healthcare. This will allow us to estimate the burden of CHE and explore gender differences by examining whether households led by men or women experience a higher risk of incurring CHE. All texts that contradict the focus on proportion of OOP as the primary outcome has been removed including on the calculation of the sample.<br><br>Terminology – gender vs. sex: We agree with the suggestion to consistently use "gender" instead of "sex," aligning the language to discuss "men" and "women." This adjustment has been made throughout the manuscript.<br><br>Manuscript updates: we have revised the manuscript to reflect these changes, including a

	clearer motivation for the gendered analysis, clarification of the definitions used, and a strategy for minimizing bias during data collection.<br><br>Methods:<br>*Specific objective 3: To include a gender equity evaluation in the study, conduct subgroup analyses within each gender to assess (1) how the UHC indigent program's impact on the level of out-of-pocket costs and catastrophic health expenditure differs between male and female led households disaggregated by gender of household members (men vs women)*<br><br>-------------------------<br><br>*In this study, we aim to explore the gendered impact of the program on household health expenditure. We define "men-led" and "women-led" households based on the self-reported gender of the household head. The household survey includes questions that ascertain the roles of household members, enabling us to distinguish between different household leadership structures accurately.*<br><br>------------------<br><br>*The primary outcome will be the proportion of OOP measured against household expenditure.*<br><br>Study limitations:<br>*Lastly, collecting accurate data on household health expenditure can be challenging. Respondents may provide inaccurate or incomplete information, leading to measurement error. Given the gender focus of our study, we recognize the potential for recall bias, especially where male household heads may not be fully aware of healthcare spending for female household members. To address this, enumerators will be trained to probe for accurate household health expenditure and, where possible, involve the household member most knowledgeable about healthcare costs in the response process.*
More care needs to be taken in the presentation of the sample size calculation on P16.<br>Please address the following:<br>• Provide a reference for where you have taken this formula from<br>• Be careful in the formatting: I suspect the "+" before Z_beta should not be subscript.<br>• In the explanation of formula terms, a number of text elements need to be made<br>• subscript (e.g. α/2). Also, "(13.1% = 0.)" and "*(8 percentage points = 0.08)*" should be corrected and made consistent.	Thank you for pointing out these issues in the presentation of the sample size calculation. We have made the necessary revisions in response to your suggestions:<br>1. **Reference for the formula**:<br>We have provided an appropriate reference for the sample size formula.<br>2. **Formatting adjustments**:<br>We have corrected the formatting of the formula ensuring the correct subscripts for all relevant terms for clarity and consistency.<br>3. **Clarification of values**:<br>The text "13.1% = 0." and "8 percentage points = 0.08" has been corrected to reflect the appropriate

• "*p = Estimated proportion of the outcome*" – the "p" needs a subscript.<br>• The bullet point for effect size specifies it as 1.2 ("DEFF = Design effect (1.2)"), while the text has this as 2 ("a design effect of 2").	values and made consistent throughout the manuscript.<br><br>The term p_1 & p_2, representing the estimated proportion of the outcome, has been updated with a subscript for both the control and intervention groups to ensure consistency with the formula.<br><br>4. **Design Effect (DE)**:<br>We have corrected the inconsistency regarding the design effect. The design effect is correctly stated as 1.2 throughout the text, aligning with the value used in the calculation.<br><br>The revised text in the manuscript now reads:<br><br>Methods<br>*We estimated that a minimum sample of 179 households per comparison group would have 80% power to detect a 15-percentage point difference in the proportion of out-of-pocket (OOP) healthcare costs as a share of total household expenditure, assuming a proportion of OOP costs of 40% in the control group, a design effect of 1.2, and a two-sided alpha level of 0.05. The initial sample size was calculated using the following formula for comparing two proportions[1]:*<br>$n = \frac{(Z_{\alpha/2} + Z_{\beta})^2 \left[p_1(1-p_1) + p_2(1-p_2)\right]}{(p_1 - p_2)^2}$<br>*Using these parameters, the sample size was estimated to be 179 households per group. Given that up to 60% of the study participants may be lost to follow-up due to refusals or the nature of the conditions they live with over one year, the sample size was adjusted by multiplying the initial estimate by* ***1/0.4*** *to account for a 40% retention rate. Additionally, to allow for oversampling, the sample size was further increased by 50%.*
Why has a design effect of 1.2 been chosen? Some justification is needed.	Given the nature of our study, where households are clustered within geographical areas, we anticipated some degree of intra-cluster correlation. However, the level of homogeneity within clusters was expected to be moderate. Therefore, a design effect of 1.2 was chosen as a conservative estimate, balancing the need for accuracy without overestimating the potential impact of clustering. We have provided a justification for the use of the stated design effect.<br><br>Methods<br>Sample size estimation<br>*The design effect (DE) of 1.2 was chosen based on typical values reported in the literature for health-related household surveys where clustering occurs. In*

	studies with moderate clustering (such as those based on households within geographic regions or communities), a design effect between 1.1 and 1.5 is often observed [65].
P21, L10-13: "We will use principal component analysis to estimate household SES scores of the households enrolled in the indigent programme." – please provide more detail of this approach – for example, what variables will be considered in the PCA? You later comment:<br>"In addition, we will disaggregate these data based on gender to determine the gender<br>equity in the households covered by the indigent programme." – again, detail is needed on how you will categorise gender – gender of the household lead?	We have revised the manuscript to provide additional detail on both aspects. Specifically:<br>1. We have now outlined the key variables that will be included in the PCA to estimate household SES, including assets, housing characteristics, and access to utilities.<br>2. We have clarified that gender will be categorized based on the gender of the household head, and we will disaggregate the SES data by men-headed and women-headed households to assess gender equity within the indigent programme.<br>These revisions can be found in the methods section as highlighted below.<br><br>Methods<br>Data analysis:<br>*We will use principal component analysis to estimate household SES scores of the households enrolled in the indigent programme. To assess the socioeconomic characteristics of households enrolled in the indigent program, we will use Principal Component Analysis. PCA is a statistical method that reduces the dimensionality of data by identifying a smaller set of uncorrelated variables, called principal components, that capture most of the variance in the original variables.*<br><br>*In our analysis, we will consider a range of variables relevant to household SES, including: 1) type of dwelling (e.g., owned, rented, shared), number of rooms, and access to basic amenities (e.g., electricity, sanitation) 2) ownership of durable goods such as televisions, refrigerators, and bicycles. This variable list is derived from the Kenya Health Equity tool (ref), which was developed for demographical health surveys, and validated for Kenya. The first principal component derived from this analysis will represent a composite measure of household SES, with higher scores indicating higher socioeconomic status in our sample. We will then map these scores against the asset score boundaries of SES quintiles of the Kenya demographic health survey 2022. The proportion of the households enrolled in the indigent program that belong to Q3-Q5 in the national socioeconomic quintiles will constitute the inclusion errors. In addition, we will disaggregate these data based on gender of the household head to determine the gender equity in the households covered by the indigent programme.*

P12, L21-26: "The SHI is expected to cover a standard benefit package in secondary and tertiary thereby guarantee indigents access to a standard package similar to contributing beneficiaries."- I'm somewhat unclear if the 'standard package' will be 'running' during the duration of this evaluation – will we know what contributing beneficiaries receive?	Thank you for your question. Yes, the same standard package of care will be accessible to all registered members, whether they are paying contributors or beneficiaries under the government subsidy program. Policy documents indicate that Social Health Insurance (SHI) system ensures that both indigents and contributing beneficiaries will have access to an identical standard benefit package. This includes secondary and tertiary care services. During the evaluation period, the standard package will indeed be operational, and we will have detailed information on the services received by contributing beneficiaries, allowing us to assess any potential differences in service utilization between the groups.
"Poverty rates within the country are estimated that nearly 40% of Kenyans [54]." – I do not believe the 'poverty' has been defined in the Kenyan setting – how is this operationalised?	National poverty rates are assessed by the national bureau of statistics-KNBC. In the Kenyan context, poverty is operationalized based on the 2021 Kenya Continuous Household Survey, as outlined in the Kenya Poverty Report 2021. Specifically, individuals or households are considered to be in overall (absolute) poverty if their total consumption per adult equivalent is less than KSh 3,947 per month in rural areas and less than KSh 7,193 per month in urban areas. This definition forms the basis for the estimation that nearly 40% of Kenyans live in poverty.
"The primary outcome will be the proportion of OOP." – please specify the denominator – household income?	Thank you for your comment. The proportion of out-of-pocket (OOP) payments will be measured against total household expenditure, which will serve as the denominator. This has been revised in the draft.
In outcomes for objectives 3, "Proportion of women included in subsidy programme" – I don't know precisely what this means – proportion of households that include women?	This is a good point. To clarify, the outcome refers specifically to the proportion of women-led households included in the subsidy program, rather than households that include women in general. This allows us to focus on evaluating the program's impact on households where women are the primary decision-makers, providing a clearer understanding of gender dynamics within the subsidy program. This has now been revised in the draft as below:<br><br>*"Proportion of women-led led included in subsidy programme"*
P19, L10-15: "Households that are not available for interviewing, either due to absence on the third attempt or a refusal, will be excluded from the survey and considered as nonresponses."<br>How does this relate to waves of the study? (e.g. "collecting data in four survey waves in 3 months intervals."). If the household is defined as non-response in wave 1, will they be approached during the subsequent waves?	Multiple contact attempts (three as a baseline) will be made, with the possibility of extending this in the initial waves. We will vary contact times and days to increase the chances of reaching households and will ensure clear communication regarding the study's importance, data confidentiality.<br><br>In order to maintain the cohort, we will only track households that we recruit at baseline. Non-respondent households from earlier waves will not be re-approached in subsequent waves. The same contact protocol from the initial wave will be

	applied consistently in subsequent waves. Additionally, we will track reasons for non-response and compare the characteristics of respondents and non-respondents across waves to assess potential biases.<br><br>The methods section has been updated as below:<br>*Household survey data will be collected through an interviewer-administered structured questionnaire within households. Interviewers will make three contact attempts as a baseline to reach the selected household respondent, with the possibility of extending these attempts in the initial waves to maximize response rates. Contact times and days will be varied to increase the likelihood of reaching households. Clear communication will be provided to explain the study's importance, data confidentiality. Households that remain unavailable for interviewing after the final attempt or refuse participation will be classified as non-responses for that wave. We will follow up households recruited at baseline. Non-respondent households from baseline will not be re-approached tin subsequent waves. The same contact protocol used in the initial wave will be consistently applied in the later waves. Reasons for non-response will be tracked, and the characteristics of respondents and non-respondents will be compared across waves to assess potential biases*
As I read BMJ Open guidance, I believe expected dates of the investigation should be provided – I don't believe these are?	Given that the intervention and its implementation are constantly evolving, including adjustments to the implementation start date, it has been challenging to provide specific expected dates for the investigation at this stage. We will continue to monitor developments and update this information as soon as more concrete timelines are established.<br><br>Study challenges and limitations<br>*The primary concern in this study is dynamic nature of UHC reforms on the implementation of the intervention. As a result, the intervention described here, and its implementation is constantly evolving including the implementation start date*
**Reviewer 2**	
**Abstract**	
Lines 30 to 35. Be precise about how recent the UHC strategy was introduced in Kenya. Same comment in the background section where you mentioned "recent".	We have now specified in the abstract and the background section that the reforms were introduced in October 2023
Page 2, line 53. You could clarify that you intend to estimate inclusion errors associated the scheme.	We have now specified in both the background and methods section that we will estimate the inclusion of the poverty targeting mechanism
**Background**	
The Act also specifies that indigent households will be	The health insurance cover provided by the government to households in the indigent program

covered through contributions from the government tax budget allocation". Does this<br>not make non-indigent household with similar characteristics suitable control group?	will be based on SES of the beneficiaries. Therefore, the cohort recruited in to the two comparison groups will need to be similar on baseline characteristics including their poverty status. Although non-indigent households might share some characteristics with the indigent group (e.g., geographic location, family size), they likely differ in unmeasured socioeconomic factors such as financial resilience, access to credit, or informal support systems. These differences could bias the results and dilute the effect of the intervention under study.
The point about social protection programs not reaching intended beneficiaries were made at least three times. I feel it is repetition and could be merged. Example,<br>page 5 lines 35, 54 to 58.	We have removed the repetition by deleting/revising areas where this appeared.
The two sentences on page 6 lines 53 to 60 contradicts each other. On one hand you say there is evidence showing the poor are disproportionately affected by CHE and the other hand, you say there is limited evidence on it.	We agree that these statements may be confusing to the reader. We have revised the statement to clarify the dearth of knowledge on the effectiveness of subsidies in reducing OOP:<br><br>"*Disaggregated data on catastrophic health expenditure (CHE) suggests that the poor are disproportionately affected by CHE. However, there is limited evidence on the effectiveness of health insurance subsidies in reducing healthcare costs and improving utilization among poor households.*"
"provide evidence of the extent of the financial protection offered by the cover on<br>indigent household" as opposed to what/who?	Thank you for your comment. Our counterfactual/comparator is indigent households that do not receive the health insurance subsidy. In our study, we are specifically comparing the out-of-pocket (OOP) healthcare costs between indigent households that are covered by the health insurance subsidy and indigent households that do not have this cover. This comparison allows us to assess the extent of financial protection provided by the subsidy within the same socio-economic group. The goal is to evaluate how well the subsidy reduces OOP expenses and protects against catastrophic health expenditure (CHE) for those receiving the cover.<br>Revision to the justification:<br>*As Kenya rolls out its UHC plans under the national social health insurance fund, it is crucial to assess the impact of the health insurance subsidy on household health expenditure among indigent households. Specifically, this study will provide evidence on the extent of financial protection by comparing out-of-pocket (OOP) healthcare costs between indigent households with the insurance cover and those without.*
How different is point 4.3 from point 4.3.1.1?	4.3 outlines the overall study design that includes- two main study designs- the cohort study and the cross-sectional. 4.3.1.1 specifies the study design of the cohort study. We have revised the draft to show the distinction.

The authors mentioned the intention to use a prospective cohort design. Is not clear how the prospective design will be applied in a household survey. In my view, prospective cohort design is more applicable at point of care.	We agree with the reviewer that the recruitment of study participants at the point of care is a common approach to recruitment into cohort studies and follow-up. However, it is also possible to use other recruitment points for a cohort study. In our case, given that the intervention under study is not a health service or clinical intervention, point of care recruitment and follow-up would not be appropriate. Further, outcomes of the study (out of pocket payments and catastrophe) are best measured at the household level. For this reason, households will be recruited from the indigent list that will be used by the social health insurance scheme to select households to that will benefit from the subsidy programme. Household surveys are used to follow up study participants.
How are the authors operationalising socio economic status as a matching criterion?	We are operationalizing socio-economic status as a household asset index based on a validated household asset index tool for Kenya. Asset ownership data will be collected and use to compute an asset index using principle component analysis (PCA).<br><br>The draft has been modified as follows in the methods section:<br>*The first specific objective of this study will employ a prospective matched cohort design with households matched using characteristics that include household size, socio economic status (asset index), and county of residence.*
.... the source documents providing a list of indigents cannot be verified....<br>Where was the list document obtained from that it cannot be verified?<br>Recast the statement to emphasize that obtaining reliable list of beneficiaries from one source may be challenging, demanding that you consult other relevant stakeholder institutions which may take some time due to bureaucratic processes of some sort.	Thank you for your suggestion. Indeed, it remains unclear who will serve as the custodians of the indigent list database, which could impact the process of identifying and enrolling study participants, but we anticipate the need for various stakeholder engagement to access the database.<br><br>We have clarified this limitation in the draft as follows:<br><br>*In addition, during the implementation of the indigent program, obtaining a reliable and verified list of indigent households may prove challenging due to uncertainties regarding which institution will act as the custodian of the indigent list database. This could impact the process of identifying and enrolling study participants. To address this, we will explore various potential sources for the indigent list, including the county department of health, the social health insurance fund, the community health assessment, and the Ministry of Labor and Social Services. It is likely that triangulating these sources will be necessary to develop a comprehensive and reliable database for recruiting study participants*

VERSION 2 - REVIEW

Reviewer 3

Name Otieku, Evans

Affiliation University of Ghana, Institute of Statistical, Social and Economic Research

Date 16-Oct-2024

COI

The authors have satisfactorily responded to my review comments. Therefore, I recommend the revised manuscript for publication.
